# Vienna Letter

**Published:** 1882-04

**Authors:** W. T. Belfield

**Affiliations:** Vienna


### Foreign Correspondence.

---

### ARTICLE IV.

VIENNA, March 6, 1882.

EDITORS CHICAGO MEDICAL JOURNAL AND EXAMINER: The leading topic of the day in German surgical circles is the place of iodoform in surgery. Six months ago the new antiseptic seemed to have supplanted all others in the treatment of wounds, whether old or recent, septic or aseptic, and to possess many and important advantages, especially over carbolic acid. An enthusiastic advocate, Mikuliez, even laid down the proposition based upon his observation and experience as Billroth's assistant, that "of the entire Listerian dressing nothing now remains except the principle." Such was indeed the case in many of the large surgical clinics, notably that of Billroth, in Vienna, and surgeons who had the remedy simply on trial, almost without exception acknowledged that, while iodoform was, in its antiseptic action, the equal of any other agent, the ease of its application to *all* wounds, the simplicity of its employment, its anæsthetic properties, and its specific influence in transforming a tuberculous, fungous surface into healthy granulations, promised its rapid and universal substitution for all other antiseptic means.

Yet this chorus of praise to the newly enthroned, malodorous goddess of antiseptic surgery was rudely interrupted some months ago by the discordant wailings of sundry of her worshippers, who lamented that while her numerous virtues were unassailable, she had at times a decided homicidal tendency. This slander was at first treated as the hallucination of a madman, but the facts accumulated so rapidly that iodoform degraded from its high estate, stands to-day a prisoner at the bar of German surgery, to plead for very existence in the surgeon's armament.

Yet the reaction is scarcely surprising when one considers the reckless way in which the agent has been used. Originally introduced by Zeissl and others as an efficient dressing for syphilitic ulcers, it was first used and brought to notice as a universal antiseptic dressing by Mosetio Moorhof in Vienna, about two years ago. In his hands it has produced no evil results, even when poured freely into wounds and cavities to any extent. Adopted and recommended to the surgical congress in Berlin last year by Billroth, and indorsed by Gussenfauer, of Prag, the remedy was soon in the hands of every enterprising surgeon. Under the impression that iodoform was perfectly innocuous, surgeons were long in the habit of applying it without stint, pouring 100, 200, even 400 grammes into a wound, so as to fill up the cavity completely. The result was many cases of severe, even fatal intoxication. Konig, of Gottingen, has recently published in the "Centralblatt für Chirurgie" thirty-two cases of more or less severe poisoning from iodoform dressing, which have been sent to him in answer to a published request for such cases. Of these he considers fifteen as slight degrees, seventeen, severe, six of the latter fatal from direct effect of iodoform. He remarks: "In the great majority of all (32) cases, much more than 10, in a large number 40, 50, 80, even 100 grammes at least were used." Yet in one case, a boy of eighteen, slight delirium, lasting a few hours, followed the application of a single gramme in the cavity of a tuberculous abscess. It is interesting to note that although in this case, the same quantity was subsequently repeatedly applied, no further abnormal symptoms were observed. This and similar cases of unusual susceptibility are explained *pro tempore* by the assumption of an "idiosyncrasy." Several points made by Konig are of great value. The poisonous effect of idoform is indicated by a rapid and feeble pulse, and by psychical disturbance—observations quite in accord with the experimental results of Binz and Högyer, who found paralysis of the heart and narcotic effects to result from exhibition of idoform in dogs and cats. Further, the dangerous forms of intoxication occur almost exclusively in the *aged*, or in the *young with organic cardiac disease;* eleven of the thirty-two patients were more than sixty years old, and *nine of the thirteen severe* (and fatal) cases were *over fifty years of age;* with children free from cardiac disease, the surgeon has very little to fear. Light degrees of iodoform intoxication are indicated chiefly by hallucinations, usually of transient (a few hours') duration; patients insist upon getting out of bed, and usually imagine themselves threatened by physical injury. The severer forms exhibit abnormal frequency and weakness of pulse, delirium, hallucination; patients refuse nourishment, become melancholy, sometimes violent; death occurs from paralysis of heart and respiration. After use of excessive quantities of iodoform, especially in children, there is sometimes observed the picture of a severe meningocephalitis—stupor, coma, involuntary passage of urine and fæces; the autopsy reveals fatty degeneration of heart, liver and kidneys; within the cranium either nothing abnormal or a chronic meningitis.

The minimum dose which can produce abnormal symptoms is not yet determined; seems to depend entirely upon the "idiosyncracy" of the patient in fact; yet König remarks that, as a rule, the danger increases with the quantity given, and finds scarcely a case of severe symptoms following the use of less than 10 grammes.

In conclusion, while König regards the use of iodoform dressings as justifiable only where antiseptic results can not be obtained by other means (especially Lister's dressing), he insists upon the use of iodoform as indispensable in the treatment of tuberculous joints, and upon mucous membranes—mouth, rectum, vagina, etc.

Billroth, however, has not the same horror of iodoform. Since April, 1881, this substance has constituted the staple dressing for *all* wounds in his immense clinic. He had last year two fatal cases of poisoning—both scrofulous children—in one case 40 grammes of crystals were poured into a large cavity in the hip after evacuation of pus—in the other, 120 grammes were placed in the wound after resection of the head of femur. Thereupon he modified the mode of application so as to reduce to a minimum the quantity applied. He now employs not crystals, but powder, which is applied to a given surface either by means of a pepper-box, or is dusted on from cotton. If union by first intention is designed, the wound is then closed with carbolized silk sutures; if small, and hence likely to discharge but little, the seam is simply covered with cotton (dusted over with iodoform), over which is laid a water-tight covering—Mackintosh for example—and a simple bandage; if the wound be large and promises profuse suppuration, iodoform gauze is applied in the same way as carbolized gauze after Lister. This iodoform gauze is very simply prepared by pouring the necessary amount of iodoform powder into a basin, and then rubbing thoroughly around in the powder, laundress fashion, a piece of suitable, large meshed cloth. In this way from ten to twenty per cent. of the weight of the bandage is found to consist of iodoform—a bandage which in Billroth's clinic has supplanted Lister's.

In mucous cavities, he uses a gauze prepared with gelatine, and containing thirty to fifty per cent., in order to compensate for the loss consequent on constant movement and irrigation of surfaces. For fistulæ in ano, for the nose, uterine cavity, etc., he makes suppositories of iodoform and gum arabic, with or without glycerine, according to the consistence required. For irrigation of mucous membranes (bladder), and for subcutaneous injections, a suspension of iodoform in glycerine (1 to 10) or in ether (1 to 5) is employed.

The first bandage is usually removed the fourth or fifth day, though sometimes allowed to remain ten, twelve, even fourteen days. Since granulations after long iodoform dressing are apt to be flabby, a stimulating salve or wash (arg. nit. 1 to 100) is usually substituted as soon as the surface is observed to be covered with granulations. Usually, two or three bandages are sufficient to secure this result.

Since iodoform is not soluble in convenient menstrua, Billroth still uses carbolized solutions for disinfection of hands, sponges, instruments, etc. Without entering into details as to results, let me say that the condition of the patient after iodoform dressing is, with rare exceptions, afebrile, painless; the wound is aseptic, secretes but little, and heals as rapidly as by any other method. Nothing could present the happy results more forcibly than the statistics of Billroth's operations for extirpation of carcinomatous tongues. Between 1871 and 1876 the mortality was thirty-two per cent.; in 1877—1880 (fifty-three cases) with irrigations of pot. permanganate solutions, eighteen per cent.; between April and October, 1881, he performed the operation eighteen times, with the same operative technique as before, but with iodoform dressing; result, 18 *recoveries*.

The course of the recovery presented in no case unfavorable symptoms. Billroth himself ascribes the extraordinary success largely to iodoform. The dressing consisted simply in a piece of iodoform gauze fifteen to twenty c.m. long, containing about one gramme of iodoform, rolled up so as to form a tampon, and pressed into the wound. The tampon was generally renewed in from five to eight days after the operation.

Mosetig is now using iodoform suspended in glycerine as a local injection in the treatment of goitre. It is safe and efficient, he says.

Neumann injects the buboes from a hard chancre with the same liquid; the glands usually become smaller and softer, but the roseola appears invariably in due course. He also gives iodoform in pills, instead of pot. iod. for constitutional syphilis.

W. T. BELFIELD.

